# Non-melanoma skin cancer diagnosis: a comparison between dermoscopic and smartphone images by unified visual and sonification deep learning algorithms

**DOI:** 10.1007/s00432-021-03809-x

**Published:** 2021-09-21

**Authors:** A. Dascalu, B. N. Walker, Y. Oron, E. O. David

**Affiliations:** 1 Department of Physiology and Pharmacology, Sackler School of Medicine, Tel Aviv University, 6 Matmon Cohen Street, 6209406 Tel Aviv, Israel; 2 Sonification Lab, School of Psychology and School of Interactive Computing, Georgia Institute of Technology, Atlanta, Georgia United States; 3 Department of Computer Science, Bar-Ilan University, Ramat-Gan, Israel

**Keywords:** Preventive medicine, Deep learning, Sonification, Non-melanoma skin cancer, Dermoscopy, Telemedicine

## Abstract

**Purpose:**

Non-melanoma skin cancer (NMSC) is the most frequent keratinocyte-origin skin tumor. It is confirmed that dermoscopy of NMSC confers a diagnostic advantage as compared to visual face-to-face assessment. COVID-19 restrictions diagnostics by telemedicine photos, which are analogous to visual inspection, displaced part of in-person visits. This study evaluated by a dual convolutional neural network (CNN) performance metrics in dermoscopic (DI) versus smartphone-captured images (SI) and tested if artificial intelligence narrows the proclaimed gap in diagnostic accuracy.

**Methods:**

A CNN that receives a raw image and predicts malignancy, overlaid by a second independent CNN which processes a sonification (image-to-sound mapping) of the original image, were combined into a unified malignancy classifier. All images were histopathology-verified in a comparison between NMSC and benign skin lesions excised as suspected NMSCs. Study criteria outcomes were sensitivity and specificity for the unified output.

**Results:**

Images acquired by DI (*n* = 132 NMSC, *n* = 33 benign) were compared to SI (*n* = 170 NMSC, *n* = 28 benign). DI and SI analysis metrics resulted in an area under the curve (AUC) of the receiver operator characteristic curve of 0.911 and 0.821, respectively. Accuracy was increased by DI (0.88; CI 81.9–92.4) as compared to SI (0.75; CI 68.1–80.6, *p* < 0.005). Sensitivity of DI was higher than SI (95.3%, CI 90.4–98.3 vs 75.3%, CI 68.1–81.6, *p* < 0.001), but not specificity (*p* = NS).

**Conclusion:**

Telemedicine use of smartphone images might result in a substantial decrease in diagnostic performance as compared to dermoscopy, which needs to be considered by both healthcare providers and patients.

## Introduction

About 5.4 million new Non-Melanoma Skin Cancers (NMSC), the most frequent skin cancer, are diagnosed each year in the US, in over 3.3 million subjects (Rogers et al. 2015). Mortality from cutaneous squamous-cell carcinoma is underreported and may approach mortality from malignant melanoma (Nehal and Bichakjian 2018). Basal cell cancer (BCC) and squamous cell carcinoma (SCC) are keratinocyte-derived skin cancers presenting with a BCC to SCC ratio of up to 4: 1. NMSC are non-melanocytic and non-pigmented in general, and therefore can be more difficult to diagnose than pigmented lesions. The number of biopsies required to diagnose a NMSC in the US ranges from 1:2 for dermatologists (Privalle et al. 2020) to 1:3 for advanced practice professionals (Nault et al. 2015). Similarly, a large scale screening intervention program in Germany indicated SCC and BCC lesion ratios needed to be biopsied to identify one NMSC was 1:4 for dermatologists and 1:9 for non-dermatologist physicians (Waldmann et al. 2012).

The use of dermoscopy—the standard of care—by physicians confers a diagnostic advantage for NMSC identification over visual inspection. A Cochrane review concluded that dermoscopy increases sensitivity of NMSC diagnostics by 14% over visual inspection (Dinnes et al. 2018). In another study with dermatologist raters, the sensitivity of NMSC diagnosis for BCC when using dermoscopy was 91%, which was 34% greater than when using close-up images; for SCC diagnosis, dermoscopic diagnosis sensitivity was 77%, which was 7% better than using close-up images (Tschandl et al. 2019). These levels of human performance leave room for improvement by a CNN usage in dermatology in to avoid unnecessary excisions or extended surgical interventions and possible disfigurement.

Due to COVID-19 restrictions on healthcare and a tendency to limit specialty clinic visits during the pandemic, telemedicine monitoring has increased; this is potentially beneficial for patients, since it might improve early diagnostics. At present, a 3-month delay in treatment of NMSC is allowed (Baumann et al. 2020), although it is known that for about half of BCC lesions that do increase in size, the mean increase in area is about 8.3 mm^2^/month (Wehner et al. 2018). SCC metastasize in about 3.7% of patients (Schmults et al. 2013) and extranodal extension diagnostics by deep learning algorithms was suggested (Kann et al. 2020). Such algorithms integrated into a telehealth setting (Kuziemsky et al. 2019) are a feasible candidate for skin cancer screening and triage (Garg et al. 2018).

Previously, we have described a dual deep learning classifier by combining a sonification layer (visual data to sound conversion) and a visual analysis layer and which reports analytics by an interpretative audio signal, a semi-supervised machine learning. This dual unified algorithm (DUA), a decision support tool for use of all physicians, improves accuracy of diagnosing skin cancer (Walker et al. 2019) and assists in clinical decisions by conveying to the physician a dichotomous prediction of lesion etiology as either benign or malign. Such algorithms might be useful as a clinical support tool for distant location diagnostics and non- dermatologists. DUA performance was validated through a controlled prospective study (Topol 2019) in a clinical environment (Dascalu and David 2019). Since our training dataset included both dermoscopic images and close-up (non-dermoscopic) photos, it is a sensible next step to assess the accuracy of NMSC diagnostic outcomes with professional dermoscopic images and non-dermoscopic smartphone images as evaluated by our DUA. Understanding the usability and effectiveness of NMSC office- or home-based diagnostics by CNN is highly relevant to the current environment, as different cancer detecting tools are being tested (Jeyaraj and Nadar 2019).

## Methods

### Primary deep learning training and sonification

As previously described14, a convolutional neural network architecture based on the Inception V2 network, a second generation CNN which uses batch normalization for classifying, was utilized. All images were validated by biopsy reports, classified into either malignant or benign and a feature representation was obtained. Publicly available datasets, such as the International Skin Imaging Collaboration (ISIC) 2017 dataset (Codella et al. 2018) and the Interactive Atlas of Dermoscopy (IAD) dataset (Lio and Nghiem 2004) were used for training to a total of 4361 advanced dermoscope images and 800 non-dermoscopic regular photos. Data augmentation, training and fine tuning were performed as previously reported, and the weighted activations of all of the 1024 nodes in the penultimate layer of the DL classifier were sonified (Walker and Nees 2011). A K-means clustering algorithm (Celebi et al 2013) was employed to cluster the activations into groups of related observations. The clustering solution with the lowest error (i.e. the one that maximizes the likelihood of the data) was chosen as the final model. Cluster centroids represented by individual pitches and malignant “alert” sounds were mapped onto loudness, timbre, and duration of a sonification, thus an audio signal for each of the centroids of data was derived, providing for an audio output that acoustically differentiated the malignant from benign lesions and conferring information about the image through a raw wave file as previously described (Walker et al. 2019).

### Unified dual deep learning algorithms

**Fig. 1:**
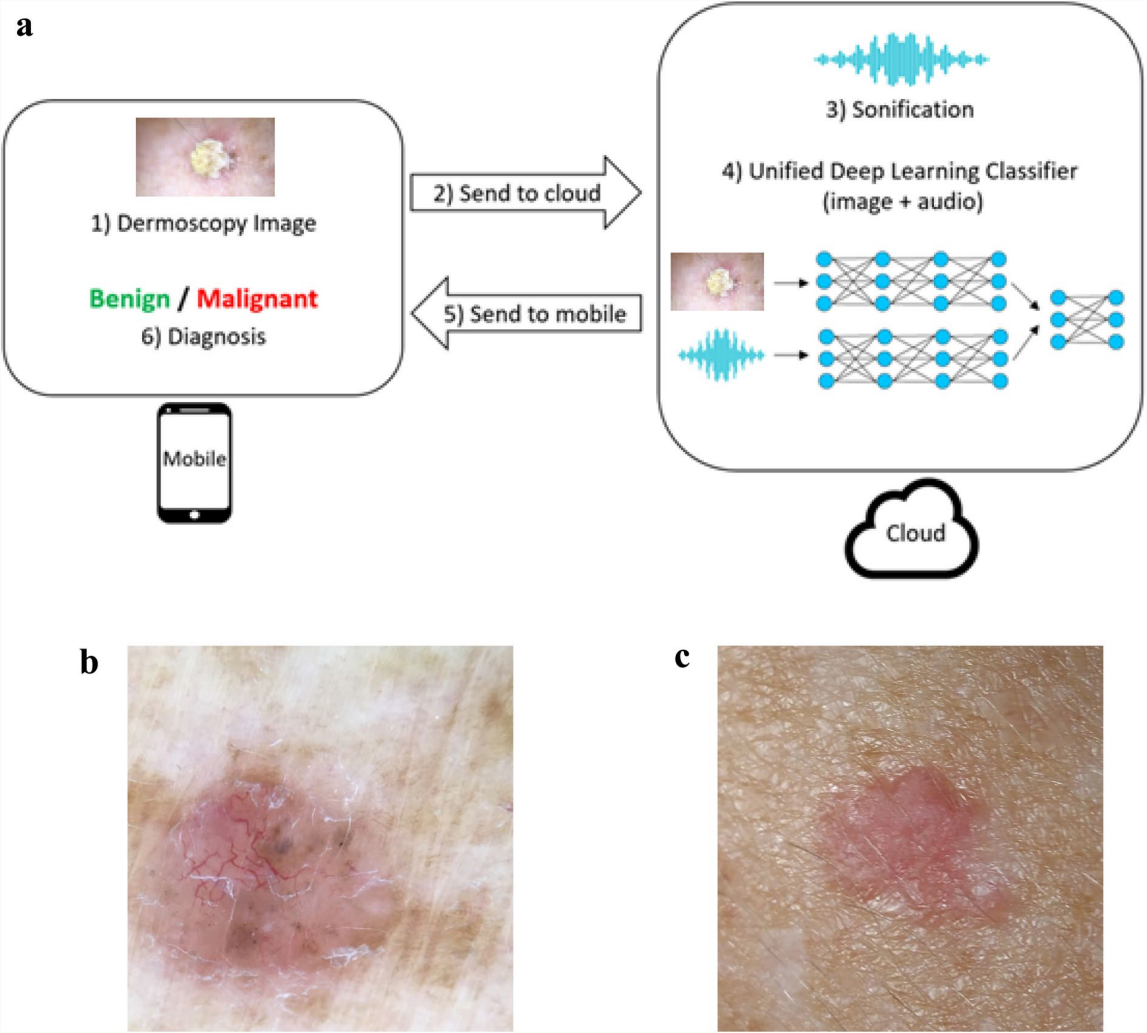
Flowchart prediction process: a dermoscopy image is acquired by a smartphone and conveyed to cloud computing by a dedicated application. A deep learning classifier and audio classifier which were pre trained are combined and predict output findings. The final diagnosis is conferred to user as a malignant or benign lesion diagnosis, i.e. excise or not indication (**a**). See basal cell carcinoma outlook by a dermoscope (**b**) or as captured by a smartphone (**c**)

Our approach is based on a combination of two independently deep learning models, which are then unified to train together at a unified threshold of 0.0101 (Fig. 1). The first deep learning model is a convolutional neural network that receives the raw image, and is trained to predict whether it is malignant or not. The second deep learning model is also a convolutional neural network which processes an audio file, which is obtained by performing sonification to the original image (in our previous works we demonstrated the benefit of sonification for this domain). Each image, either dermoscopic or smartphone, was processed by the same methodology, i.e. by both a raw image classifier and independently by a sonification classifier.

To further improve the accuracy, we then combine the two models as follows: the output (prediction) layer of both deep learning models is removed, such that the prior layer is not the output layer. Two new fully connected layers are then added on top of the two models, such that the outputs of the two models are the inputs to the fully connected layers. A new softmax prediction layer is added to the fully connected layers, which provides the final unified prediction. Then, this entire unified structure is trained until convergence. Most of the training takes place in the fully connected layers, whereas the prior layers which are pretrained are only fine-tuned. The DUA takes advantage of raw image and sonification algorithms differential advantages in increasing specificity and sensitivity, correspondingly (Walker et al. 2019). The recommendation of the unified system is dichotomous and relates to decision making, either an excise or do not excise, which conforms to a physician diagnostic decision and avoids mixing a specific diagnosis with pathological considerations, as remarked for other oncologic areas of interest (Kann et al. 2020; Simon et al. 2020).

The unified output was applied on histopathology validated NMSC images of dermoscopic (Fig. 1a) or smartphone origin (Fig. 1b).

### Datasets analysis

The dermoscopic images were captured by dermatologists or trained GPs by digital and smartphone camera and derived from two sources (Table 1), the Ham10000 database (Tschandl et al. 2018) applying a 1:5 randomly selection (*n* = 149) and our previous prospective clinical study (Dascalu and David 2019) (clinicaltrials.gov Identifier: NCT03362138, *n* = 16). Both polarized and non-polarized dermoscopic images were included. Non-dermoscopic smartphone images, captured by different non-standardized smartphone devices (Table 1), were derived from a recently published and biopsy-validated dataset (Pacheco et al. 2020) dedicated to NMSC at a 1:3 random selection (*n* = 159) and The Journal of Investigative Dermatology Editorial Images 2018 (*n* = 39). Random selections were performed by using a Phyton script.

**Table 1:** Epidemiologic data and characteristics of lesions

Dermoscopic images characteristics	
Age, mean (range)	67.2 ± 12.3 (31–87)
Sex	
Male	107
Female	58
All images histopathology diagnosis	165
BCC	96
SCC	36
Seborrheic Keratosis	33

Non-dermoscopic images characteristics	
Age, mean (range)	66.3 ± 14.3 (22–91)
Sex	
Male	134
Female	64
All images histopathology diagnosis	198
BCC	139
SCC	31
Seborrheic Keratosis	28

Patient characteristics by age (*p* = 0.53, NS, student’s *t* test) and gender (*p* = 0.58, NS, Chi squared test) are without a difference

All dermoscopic and smartphone datasets were triaged by an expert dermatologist (AD) applying exclusion criteria identical to our previous prospective clinical study. Images displaying whole organ appearance, ink markings, more than 15 hairs per field, scale bars extraneous to the capturing device, blurred images and photos less than 200 Kb (omitted due to a marginal resolution conferred by low pixels, about 36% of omitted images) were excluded post selection. Exclusion rates were similar, i.e. 16.6% and 18.7%, for dermoscopic and non-dermoscopic images, respectively. Dermoscopic images of Ham and JID dataset did not include ethnicity details, precluding such comparisons between datasets. Seborrheic keratosis lesions with enough criteria to be excised as suspected NMSC were confined to the benign definition of the study. Actinic keratosis were not included because these lesions are a gray zone definition and a pre-neoplastic entity. An unknown fraction of these lesions are treated by consensus before excision (cryotherapy, fluorouracil use, etc.) resulting in distorted morphological features such as hyper or hypo pigmentation and scarring of the lesion and therefore were omitted from this study due to the lack of criteria of excision (Dréno et al. 2014) or any documentation details in both databases. Dermatofibromas, vascular lesions, and pigmented nevi were not included due to their diagnostic obviousness or major pigmentary nature.

### Outcomes

Primary outcome measures to compare between dermoscopic and non-dermoscopic techniques for measure by our AI algorithm score were sensitivity (sensitivity is the percentage of correctly diagnosed malignancies, i.e., true positive/positive diagnoses) and specificity (specificity is the percentage of correctly identified NMSC, i.e., true negative/negative diagnoses). Guidelines provided by Cochrane reviews which tested sensitivity and specificity at a fixed cutoff point of 80% for both parameters were used to compare dermoscopy versus eye inspection. Since ROC curves are a continuous-scale display, a cut-off point is chosen to allow comparisons between different studies output, such as a typically fixed value of 80% for sensitivity or specificity. This framework of reference was used in the present study by our DUA which replaced the human component in diagnostics by either dermoscopy or visual inspection.

### Statistical analysis

Baseline and demographic characteristics were summarized by standard descriptive summaries. All statistical tests used in this study were 2-sided and a *p* value < 0.05 was considered significant (SigmaPlot v10.0, Systat Software, SanJose, CA). Diagnostics of methodologies were quantified by the area under the curve (AUC) of the receiver operating characteristic curve (ROC) for the malignancy scores as compared to ground truth. Sensitivity, the true positive rate, was plotted on the y-axis versus [1-Specificity], the false positive rate, on the x-axis of ROC curves. AUC for such a plot has a maximum value of 1.0, and is a standard performance metric in the machine learning literature. Negative Predictive Value (NPV) are a metrics of true negative/(true negative + false negative) data and represent how likely it is for a normally tested subject to truly be healthy, in case of a negative test result. Accuracy of the ROC is defined as the fraction of correct predictions, i.e. true positives and true negatives divided by all true and false positives and negatives. A confusion matrix was used to label the performance of our classification model on each of the dermoscopic and non-dermoscopic groups.

## Results

Epidemiologic data and lesion characteristics of dermoscopic and smartphone photographs datasets of NMSC are specified in Table 1. Age and gender of the two groups were comparable between groups (*p* = 0.53, NS, student’s *t* test and *p* = 0.58, NS, Chi squared test, respectively). The histopathology-validated images were analyzed by our DUA and a particular malignancy score was derived for each image. These malignancy score of the NMSC were parsed on a scale from 0–1 (1.0 was labeled as the highest malignancy score) and dermoscopic and smartphone datasets were further depicted as a ROC curve.

**Fig. 2:**
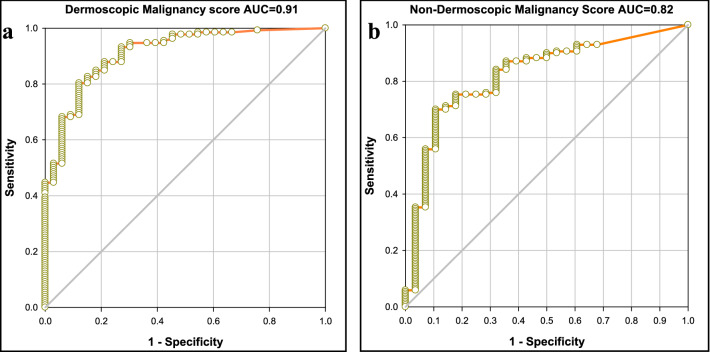
ROC curves of prediction sensitivity and specificity of the deep learning model for (**a)** dermoscopic images and (**b)** smartphone

ROC curve analytics of the dermoscopic dataset (Fig. 2a) indicated an AUC of 0.911 (95% CI 0.858–0.964). The ROC curve for smartphone-acquired dataset was further calculated (Fig. 2b) and resulted in an AUC of 0.821 (95% CI 0.738–0.905). Both AUC’s are solid but the discriminative power and overall diagnostic accuracy of dermoscopic imaging outperforms the smartphone diagnostics.

**Fig. 3:**
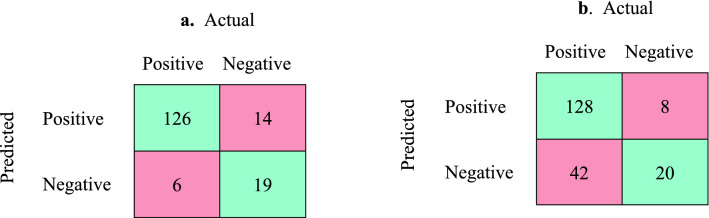
Confusion matrix for malignant versus benign lesions: **a** dermoscopic images; **b** smartphone images. Green represents the right prediction by model, red reads model was wrong

Data were further evaluated by a confusion matrix (Fig. 3) for both dermoscopic and smartphone images. A detailed classification discriminative measure was calculated as specified in Table 2 and various clinical decision metrics were compared between the dermoscopic and smartphone-acquired methods (unpaired Student’s *t* test). Overall, the accuracy (i.e., correct predictions) of DI diagnostics (87.8%) was superior to SI diagnostics (74.8%; *p* < 0.005). Upon parsing the results, the DI diagnostics yielded a higher sensitivity (95.5%) than SI images (75.3%; *p* < 0.001). Consequently, the negative predictive value of the dermoscopic images outperformed the smartphone images (*p* < 0.001). The positive predictive values for both smartphone and dermoscopy are adequate and at the highest range of a clinical spectrum of accuracy (i.e., 90 + %). However, the low negative predictive values for the smartphone images reflect a missed NMSC in about 25% of lesions (42/170) which severely restricts its predictive value for any negative diagnosis of NMSC.

**Table 2:** Metrics of diagnostic analysis of images acquired through a dermoscopic lens versus smartphone

Metrics	Dermoscopy (95% CI), %	Smartphone photo (95% CI), %	*p*
Sensitivity (recall), TP/(TP + FN)	95.5 (90.4–98.3)	75.3 (68.1–81.6)	*p* < 0.001
Specificity, TN/(TN + FP)	57.6 (39.2–74.5)	71.4 (51.3–86.8)	*p* = 0.28 NS
Precision, TP/(TP + FP), positive predictive value	90.0 (85.8–93.1)	94.1 (89.9–96.7)	*p* = 0.73 NS
Negative predictive value TN/(TN + FN)	76.0 (57.9 to 87.9)	32.3 (25.1–40.4)	*p* < 0.001
Accuracy (TP + TN)/(TP + TN + FP + FN)	0.878 (81.9–92.4)	0.748 (68.1–80.6)	*p* < 0.005

The values from Fig. 3 were used to derive data of this table

*TP* true positive, *TN* true negative, *TP* true positive, *FP* false positive

We further measured the outcome of NMSC evaluation by substituting AI assessment for human diagnostics. Criteria similar to a recent Cochrane review were applied to our data, namely applying fixed cutoffs of 80% for either sensitivity or specificity, and testing the outcome for the non-fixed parameter. NMSC diagnosis performance was predicted on the relevant ROC curve as follows: (i) Postulating a fixed specificity of 80%, the sensitivity was 85% for dermoscopy versus 76% for smartphone (dermoscopy advantage =  + 9%); (ii) at a fixed sensitivity of 80% the specificity was 88% for dermoscopy and 69% for smartphone (dermoscopy advantage =  + 19%). These figures are in concordance and close to human performance, which present a dermoscopy advantage of + 14% for a fixed specificity and + 22% for a fixed sensitivity(Dinnes et al. 2018).

## Discussion

A dual CNN was used to compare skin cancer diagnostics of dermoscopic versus smartphone camera images. It is demonstrated that DI are detected at a high sensitivity of 95% and specificity of 58%, a PPV of 90% and a NPV of 76%. Settling for SI, overall accuracy of the system drops by about 13%, sensitivity is 20% lower and NPV subsides at 32%. It was reviewed that dermoscopic visualization by human assessment outperforms clinical naked eye inspection in face to face encounters (Dinnes et al. 2018) and telehealth consultations (Ferrándiz et al. 2017). We conclude that a proclaimed gap in diagnostic accuracy between dermoscopy and clinical examination, as exemplified by smartphone images, is not narrowed down by use of CNN algorithms and therefore, use of a dermoscope substantially improves diagnostic accuracy with or without a CNN.

Skin cancer detection through telemedicine channels is in agreement with histopathology in about 70% of diagnoses by dermatologists (Giavina-Bianchi et al. 2020) and 50% by primary care physicians (Bridges et al. 2019). These moderate performance levels leave room to further improvements, possibly by artificial intelligence-enhanced methods. The professional patient-oriented telemedicine diagnostics at present relies on a non-standardized smartphone camera to capture an image. Unsurprisingly, a recent review concluded that algorithm-based smartphone apps are currently non-reliable (Freeman et al. 2020) and that test performance is expected to be poorer when applications are used in real life scenarios. Our study points to the image source as a place to start when seeking improvements in this domain.

Dermoscopic images are magnified by an achromatic lens ten-fold (10×) and include topographical details and microstructures down to the level of papillary dermis, unlike macro or smartphone images of skin lesion. Notably, a dermoscopic image is assessed by a limited and fixed number of dermoscopic patterns (Fargnoli et al. 2012). The improved diagnostics through the use of a dermoscope is not a straightforward conclusion since the human eye in both instances perceives a two dimensional image, which cannot be reconstructed into a reliable 3-D topographical image by a human cortex. Distinctive colors and hues of lesions do not appear to make a large difference of NMSC identification, unlike melanoma, since image acquirement of lesions by both techniques is colored and, in addition, specific color characteristics are minor criteria of NMSC dermoscopy. This does not hinder human diagnostics by dermoscope, since human heuristics work by ignoring part of the information which leads to more accurate judgments than weighting and adding all information (Gerd and Gaissmaier 2011).

We demonstrated in the past that an increase in resolution by use of a high-end device is not a critical factor of improving sensitivity since both a professional high resolution and a rudimentary grade dermoscope possess, perhaps unexpectedly, the same sensitivity (Dascalu and David^ 2019^) Similar to human heuristics—but by different mechanisms—CNNs improve specificity, but not sensitivity upon image capture by a higher-end device as compared to a low-resolution SI. It is assumed the rules of AI diagnostics are different and unrelated to human cognition-based diagnostics. The improved resolution of the dermoscope is secondary in enhancing diagnostics to the uncovering of sub-epidermal aspects of critical anatomical-pathological features conveyed by dermoscopy. Therefore, our present study emphasize an essential dissimilarity in image features between dermoscopy and regular upper surface smartphone imaging as processed by a CNN.

Study limitations include a retrospective design and comparison between different groups of patients. An ideal design will prospectively assess the same patient by both dermoscopy and smartphone, and compare with histopathological report. Although this setup is ideal, its physical implementation during the COVID-19 era is challenging, and based on the size and curation of both our testing arms of this study we assume such test results would not differ materially from the present study. A ratio of 5–7 NMSC to benign lesions was derived due to the interrelated content of the employed databases, and an increase in sample size is not expected to change these ratios. Due to a high variability of the specificity data of our sample, DI and SI specificity comparisons are precluded. Present article is relevant to sensitivity of detection of NMSC, and less to an evaluation of a larger spectrum of lesions, such as vascular structures, skin benign tumors and actinic keratosis. An additional limitation is the comparison of NMSC versus benign seborrheic keratosis with dermoscopic or visual features severe enough to require excision. We believe that by inclusion of only difficult-to-diagnose benign lesions (Papageorgiou et al. 2018), the safety of conclusions to be derived from the study is increased. This is a scientifically conservative design which trades an increase in overall sensitivity by the inclusion of obvious vascular or pigmented lesions to a more robust and real life outcomes. Finally, we included only lesions that were subjected to excision, solely, to increase accuracy. Consequently, our study is prone to confirmation bias, which leads to possible overrepresentation of benign lesions that are difficult to diagnose. To prevent confirmation bias, inclusion of lesions without ground truth is required, affecting accurate diagnostics, which we avoided.

In conclusion, a CNN employing combined visual and sonification algorithms was tested to identify NMSC amongst a set of difficult to diagnose lesions. Results indicate that CNN assessment of dermoscopic images improves NMSC diagnostics as compared to smartphone imaging, emphasizing the advantage of dermoscopy over smartphone image-based telemedicine. The use of CNN analytics does not close the already known gap in face-to-face diagnostic accuracy between dermoscopy and smartphone photos, which seems to be constitutional to the skin layer analyzed by the classifiers. Physicians and patients should be aware of a possible decrease in sensitivity whenever diagnosing by non-standardized smartphone teledermatology.

## Data Availability

Publicly created datasets for images are available. ISIC images dataset is available at ISIC Archive, https://www.isic-archive.com/#!/topWithHeader/onlyHeaderTop/gallery?filter=%5B%5D. The smartphone images dataset is available at https://data.mendeley.com/datasets/zr7vgbcyr2/1. The code for CNN is not publicly available.
